# Association of time to groin puncture with patient outcome after endovascular therapy stratified by etiology

**DOI:** 10.3389/fnagi.2022.884087

**Published:** 2022-10-10

**Authors:** Yiran Zhang, Lan Hong, Yifeng Ling, Lumeng Yang, Siyuan Li, Xin Cheng, Qiang Dong

**Affiliations:** ^1^Department of Neurology, National Center for Neurological Disorders, National Clinical Research Centre for Aging and Medicine, Huashan Hospital, Fudan University, Shanghai, China; ^2^State Key Laboratory of Medical Neurobiology, Fudan University, Shanghai, China

**Keywords:** acute ischemic stroke, endovascular treatment, time to treatment, collateral circulation, perfusion imaging

## Abstract

**Background:**

Randomized clinical trials and large stroke registries have demonstrated a time-dependent benefit of endovascular treatment (EVT) in patients with acute ischemic stroke (AIS) due to large vessel occlusion (LVO). The aim of this study was to investigate whether this could be applied to different stroke subtypes in a real-world single-center cohort.

**Materials and methods:**

Consecutive ischemic stroke patients with LVOs presenting within 24 h after symptom onset were prospectively registered and retrospectively assessed. Baseline multimodal imaging was conducted before EVT. Independent predictors of functional independence [90-day modified Rankin scale (mRS), 0–2] and any incidence of intracranial hemorrhage (ICH) were explored using a stepwise logistic regression model in the entire cohort and in stroke subtypes.

**Results:**

From 2015 to 2020, 140 eligible patients received EVT, of whom 59 (42%) were classified as large artery atherosclerosis (LAA)-related. Time from last known normal to groin puncture was identified as an independent predictor for functional independence in patients of cardioembolic (CE) subtype [odds ratio (OR) 0.90 per 10 min; 95% CI 0.82–0.98; *P* = 0.013] but not in the LAA subtype and the whole cohort. Groin puncture within 6 h after the time of last known normal was associated with a lower risk of any ICH in the whole cohort (OR 0.36, 95% CI 0.17–0.75, *P* = 0.007). Sensitivity analysis of patients with complete imaging profiles also confirmed the above findings. Besides, compared with patients of the CE subtype, the LAA subtype had a smaller baseline ischemic core volume, a better collateral status, a slower core growth rate, and a numerically smaller final infarct volume.

**Conclusion:**

Faster groin puncture has a more pronounced effect on the functional outcome in patients of CE subtype than those of LAA subtype. Reducing time to groin puncture is of great importance in improving the prognosis of patients after EVT, especially those of CE subtype, and reducing the incidence of any ICH in all patients.

## Introduction

Endovascular treatment (EVT) has become a routine clinical practice in the early management of acute ischemic stroke (AIS) due to large vessel occlusion (LVO). With the assistance of advanced imaging, patients with a favorable imaging profile can be treated with EVT up to 24 h after the time of last known normal (LKN) (Albers et al., 2018; Nogueira et al., 2018). Previous studies have demonstrated a strong time dependency on treatment benefits. With every hour saved for the time from onset to groin puncture, there is an absolute increment of the probability of functional independence by 3.4–5.3% (Saver et al., 2016; Mulder et al., 2018; Jahan et al., 2019), an absolute decline of the risk of symptomatic intracranial hemorrhage (ICH) by 0.88% (Jahan et al., 2019), and an absolute decrease of mortality at 90 days by 5.3% (Mulder et al., 2018). This time-dependent relationship exists across both randomized clinical trials (RCTs) (Saver et al., 2016) and registry studies (Mueller-Kronast et al., 2017; Mulder et al., 2018; Jahan et al., 2019), conservative therapeutic time window (Saver et al., 2016; Mulder et al., 2018; Jahan et al., 2019), and emerging tissue window (Mundiyanapurath et al., 2017; Snyder et al., 2020).

Nevertheless, these studies (Saver et al., 2016; Mueller-Kronast et al., 2017; Mundiyanapurath et al., 2017; Mulder et al., 2018; Jahan et al., 2019; Snyder et al., 2020) are limited to the Western population, among whom the prevalence of large artery atherosclerosis (LAA) is much lower than their Asian counterparts (Kim and Kim, 2014). Accumulating evidence has suggested that LAA-related stroke has a distinct etiology and pathophysiology from cardioembolic (CE) stroke. Unlike the abrupt onset of cardiac embolism, patients with LAA are subjected to a chronic period of hemodynamic instability during which collaterals have been recruited (Liebeskind et al., 2011), compensating for the deficient blood supply and preserving more salvageable tissue at the time of treatment (Kim et al., 2009). Based on the finding that ischemic core growth rate is dependent on collateral status (Lin et al., 2021), it is thus assumed that patients with LAA-related stroke may be less time-sensitive in terms of the benefit of EVT.

To address this hypothesis, we present here a real-world single-center experience of EVT from China, investigating the association of time to groin puncture with functional outcome after endovascular therapy stratified by two major stroke subtypes, namely, LAA and CE.

## Materials and methods

### Study population

Consecutive patients with AIS presenting within 24 h of LKN at Huashan Hospital between April 2015 and December 2020 were recruited prospectively for the institutional stroke registry. Written informed consent was obtained from all participants, and the study was approved by the Huashan Ethics Committee (No. 2013002).

At our institution, patients with suspected AIS presenting within 24 h of LKN routinely underwent emergent multimodal imaging studies including non-contrast computed tomography (NCCT), CT angiography of the head and neck, and perfusion CT, if there were no contraindications to the contrast agent. Candidacy for EVT was conformed to the latest Chinese guidelines and high-quality evidence (Albers et al., 2018; Nogueira et al., 2018). Endovascular intervention may consist of a simple angiography, stent thrombectomy maneuver, angioplasty, or a combination of the above. Further details regarding EVT candidacy and treatment are provided in the Supplementary material.

In this study, patients were included if they met the following criteria: (1) those had LVO or severe stenosis in the anterior cerebral circulation, confirmed by CT angiography (e.g., extracranial or intracranial segment of internal carotid artery and M1/M2 segment of the middle cerebral artery and anterior cerebral artery; severe stenosis was defined as ≥ 70% lumen narrowing); (2) aged ≥ 18 years; and (3) underwent EVT upon admission (i.e., entry into the angiography suite and initiation of groin puncture). Patients were excluded if they (1) had no CT scan within 2 weeks after EVT; (2) lost to 90-day follow-up; and (3) had missing data on hospital arrival time or groin puncture time.

### Data collection and imaging assessment

Demographic data, medical history, stroke subtypes, imaging features, procedural time metrics, and treatment details were recorded. Stroke subtypes were determined using the Trial of ORG 10172 in Acute Stroke Treatment (TOAST) classification (Adams et al., 1993). Patients with atrial fibrillation were grouped into the LAA subgroup if there was a fixed focal stenosis greater than 50% after thrombus retrieval (Jia et al., 2018), multiple/multistage ischemic lesions in a single internal carotid artery (ICA) territory, contrast-enhanced plaque upon high-resolution magnetic resonance imaging (MRI), or a previous history of stereotyped ischemic attacks (Zotter et al., 2021).

Imaging features derived from baseline CT, CT angiography, perfusion CT, digital subtraction angiography, and postprocedural NCCT were independently assessed by 2 neuroradiologists (YZ and LH). A third experienced neuro-interventionist (YL) was consulted in cases of discrepancy. Perfusion CT was post-processed by the commercial software MIStar (Apollo Medical Imaging Technology, Melbourne, Victoria, Australia) with singular value deconvolution with delay and dispersion correction. Infarct core (relative cerebral blood flow < 30%) and acute hypo-perfused lesion [delay time (DT) > 3 s] were defined according to the previously validated thresholds (Bivard et al., 2014, 2017). Penumbra volume was calculated by subtraction of infarct core volume from acute hypoperfused lesion volume. The volume ratio of DT > 6 s/DT > 3 s was used to quantify collateral status (Hong et al., 2019), with a lower DT > 6 s/DT > 3 s ratio indicating better collateral flow. The collateral index ratio was deemed zero if the volume of DT > 3 s was equal to zero. Successful recanalization was determined by the modified thrombolysis in cerebral ischemia (mTICI) score of 2b or 3 on the final angiography run. The core growth rate was estimated by the core infarct volume on baseline perfusion CT divided by the time from LKN to perfusion imaging (Vagal et al., 2018; Lin et al., 2021). Follow-up MRI scans were obtained preferably within 3–7 days after EVT and were semi-automatically measured for the final infarct volume using the MIStar Region of Interest (ROI) tool by 2 neuroradiologists (YZ and LH) blinded to recanalization grade. In cases where follow-up MRI diffusion-weighted imaging was unavailable, follow-up NCCT was used as an alternative. If present, hemorrhagic transformation was incorporated in the final infarct volume.

The presence of ICH on CT was determined by an NCCT scan routinely performed 24-h post-procedure or anytime when a clinical deterioration was observed. A repeated CT scan was performed to distinguish petechial hemorrhage from contrast staining when there was uncertainty over a postprocedural hyperdensity on NCCT. Symptomatic ICH (sICH) was defined according to the Second European-Australasian Acute Stroke Study (ECASS-II) criteria (Hacke et al., 1998).

### Study outcomes

The primary outcome was functional independence, defined as a modified Rankin scale (mRS), 0–2 at 90 days. The 90-day follow-up was assessed *via* telephonic interview by a trained nurse who was blinded to the clinical data. The secondary outcome was any incidence of intracranial hemorrhage post-EVT.

### Statistical analysis

All statistical analyses were performed on Stata/SE 15.1 (StataCorp, College Station, TX, USA). Mean and standard deviation (SD) were used to describe continuous variables if normally distributed; otherwise, median and interquartile range (IQR) were displayed. Frequency and percentage were used to describe categorical variables. Differences in baseline characteristics were compared using Student’s *t*-test or Wilcoxon rank-sum test for continuous variables, and chi-squared or Fisher’s exact test for categorical variables. Variables with *P* < 0.05 in the univariate analyses or with clinical relevance [age, baseline National Institute of Health Stroke Scale (NIHSS) score, sex, successful recanalization, and for the imaging cohort, infarct core (Campbell et al., 2019) and final infarct volume (Boers et al., 2019)] were entered into a stepwise logistic regression analysis with a removal probability of 0.05. Adjusted odds ratios (ORs) with their 95% CIs were presented. To adjust for imaging features and account for potential selection bias caused by contraindication to perfusion study or follow-up imaging scan, a sensitivity analysis was conducted in patients with complete imaging data. Additional analysis was restricted to patients with pure LVO to exclude the effect of residual anterograde collateral flow in patients with severe stenosis. A two-sided *P* < 0.05 was considered statistically significant.

## Results

Of 191 patients with AIS receiving EVT at our tertiary stroke center between April 2015 and December 2020, 140 (92.7%) were included in the primary analysis (Supplementary Figure 1), of whom 129 had pure LVO. The median (IQR) age was 71 (61–78) years with a mean (SD) baseline NIHSS score of 15 (6). Patients arrived at the emergency room with a median (IQR) of 159 (78–300) min after LKN, and the median (IQR) door to puncture time was 159 (126–191) min. The median (IQR) LKN to puncture time was 337 (235–480) min with 79 (56%) patients being punctured within 6 h. A total of 57 (41%) patients achieved functional independence (mRS, 0–2) at 90-day follow-up and any ICH occurred in 54 (39%) patients of whom 18 (13%) had sICH according to ECASS-II criteria (Hacke et al., 1998). Baseline demographic, clinical, and imaging data of patients with mRS of 0–2 and 3–6 were listed in the Supplementary material (Supplementary Table 1).

A total of 59 (42%) patients were classified as LAA subtype according to the TOAST criteria, whereas 56 (40%) patients were diagnosed as CE subtype. Patients in the LAA group were younger and had a milder symptom. A significantly higher proportion of patients was treated with EVT outside the 6-h time window in the LAA group with a more delayed LKN to puncture time. However, there were no significant differences regarding the rate of successful recanalization, functional independence, and any ICH between the two groups (Table 1).

**TABLE 1:** Characteristics and outcomes of patients with LAA vs. CE.

	LAA (*n* = 59)	CE (*n* = 56)	*P*-value
**Demographics**			
Age, median (IQR)	67 (12)	72 (10)	**0.03**
Female	17 (29%)	30 (54%)	**0.01**
NIHSS, mean (SD)	12 (8, 17)	17 (14, 19)	**< 0.001**
**Medical history**			
Smoking	26 (45%)	13 (24%)	**0.02**
Hypertension	42 (71%)	35 (62%)	0.32
Atrial fibrillation	8 (14%)	40 (71%)	**< 0.001**
Diabetes mellitus	17 (29%)	19 (34%)	0.55
Stroke	16 (27%)	11 (20%)	0.34
Antiplatelet	8 (14%)	12 (21%)	0.27
Statin	8 (14%)	3 (5%)	0.13
**Imaging features**			
Occlusion site			0.25
M1	33 (56%)	40 (71%)	
M2/ACA	3 (5%)	4 (7%)	
ICA	17 (29%)	9 (16%)	
Tandem	6 (10%)	3 (5%)	
**Treatment details**			
General anesthesia	28 (47%)	29 (52%)	0.64
IVT	23 (39%)	29 (52%)	0.17
LDT (min), median (IQR)	213 (127, 502)	96.5 (49.5, 234.5)	**< 0.001**
DPT (min), median (IQR)	165 (126, 212)	141 (120, 174.5)	**0.05**
LPT (min), median (IQR)	368 (305, 625)	247.5 (191.5, 386.5)	**< 0.001**
LPT within 6 h	28 (47%)	39 (70%)	**0.02**
**Technical efficacy**			
mTICI ≥ 2b	42 (71%)	37 (66%)	0.55
**Outcome**			
90 days mRS, 0–2	29 (49%)	22 (39%)	0.29
Any ICH	22 (37%)	22 (39%)	0.83
sICH-ECASS-II	9 (15%)	10 (18%)	0.71
90 days mortality	8 (14%)	15 (27%)	0.08

LAA, large artery atherosclerosis; CE, cardioembolism; LDT, last known normal to hospital arrival time; LPT, last known normal to puncture time; DPT, door to puncture time.

Multivariate logistic regression showed that for the entire cohort, younger age, successful recanalization, and absence of any ICH but no time to groin puncture were independent predictors of 90-day good functional outcome (Table 2). However, for patients punctured within 6 h after the time of LKN, every 10-min delay from hospital arrival (door) to puncture was associated with a 16% decline in the OR of functional independence (OR 0.84; 95% CI 0.74–0.96; *P* = 0.008; Table 2).

**TABLE 2:** Predictors of functional independence (mRS, 0–2) at 90 days.

	OR (95% CI)	*P*-value
**All patients (*n* = 140)**		
Age	0.94 (0.91, 0.98)	0.001
mTICI ≥ 2b	3.27 (1.32, 8.06)	0.001
Any ICH	0.12 (0.04, 0.31)	< 0.001
**Patients punctured within 6 h after LKN (*n* = 79)**		
NIHSS	0.86 (0.76, 0.96)	0.011
mTICI ≥ 2b	4.80 (1.30, 17.67)	0.018
Any ICH	0.06 (0.01, 0.28)	< 0.001
DPT^†^	0.84 (0.74, 0.96)	0.008
**LAA patients (*n* = 59)**		
Age	0.91 (0.86, 0.97)	0.002
Any ICH	0.17 (0.04, 0.67)	0.011
**CE patients (*n* = 56)**		
Age	0.85 (0.77, 0.95)	0.004
Any ICH	0.01 (0.00, 0.19)	0.002
LPT^†^	0.90 (0.82, 0.98)	0.013
Diabetes mellitus	0.08 (0.01, 0.69)	0.021

^†^Odds ratios are scaled per 10 min of delay in the listed intervals. LPT, last known normal to puncture time; DPT, door to puncture time.

**FIGURE 1:**
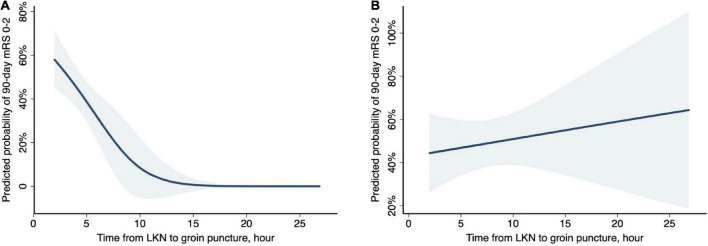
Probability of functional independence (90-day mRS, 0–2) by time from last known normal (LKN) to groin puncture in stroke subtypes. Curves (blue shading indicates 95% CIs) represent the predicted probabilities of functional independence under logistic regression models. **(A)** In CE-related strokes, after adjustment for age, baseline NIHSS, diabetes, and any ICH, there was a 47% decreased probability of functional independence per hour treatment delay (aOR, 0.53; 95% CI, 0.32–0.88; *P* = 0.013). **(B)** In LAA-related strokes, after adjustment for age, baseline NIHSS, and any ICH, the association between LKN to puncture time and functional independence did not reach a statistical significance (aOR, 1.05; 95% CI, 0.91–1.21; *P* = 0.528).

An explorative analysis revealed a significant association between LKN to groin puncture time (LPT) and functional independence (OR 0.94; 95% CI 0.89–1.00; *P* = 0.04) as well as a significant multiplicative interaction between stroke subtypes and LPT (*P* = 0.031) when restricting patients to LAA and CE subgroups. Subgroup analyses were subsequently performed. In the subset of patients diagnosed with CE-related stroke, a shorter LPT (OR 0.90; 95% CI 0.82–0.98; *P* = 0.013; Table 2 and Figure 1A) was identified as an independent predictor of a good outcome, as well as younger age, absence of the history of diabetes mellitus, and absence of any ICH. Nevertheless, in patients with LAA-related stroke, the association of LPT and clinical outcome did not show a statistical significance (OR 1.01; 95% CI 1.00–1.01; *P* = 0.635; Table 2 and Figure 1B).

In addition, LPT within 6 h was also independently associated with a lower risk of any ICH [OR 0.36; 95% CI (0.17–0.75); *P* = 0.007; Supplementary Table 2] in the entire cohort.

For the sensitivity analysis confined to patients with complete imaging data, 23 patients were excluded from the primary study (Supplementary Figure 1), while similar results were shown for a logistic regression model on 90-day functional outcome and any ICH (Supplementary Tables 2, 3). In addition, the imaging cohort demonstrated two distinct imaging profiles classified as LAA and CE stroke subtypes. Compared with patients of CE subtype, patients of the LAA subtype had a smaller baseline ischemic core volume [median (IQR), 5 (1, 22) vs. 11 (5, 27) ml, *P* = 0.044], a better collateral status [DT6/DT3, median (IQR), 0.17 (0.08, 0.38) vs. 0.31 (0.15, 0.45), *P* = 0.016], and a slower core growth rate [median (IQR), 1.2 (0.3, 3.6) vs. 4.9 (2.0, 9.8) ml/h, *P* < 0.001]. On follow-up imaging, a numerically smaller final infarct volume was shown in patients of the LAA subtype [median (IQR), 13.5 (3.8, 79.9) vs. 33.8 (13.3, 97.3) ml, *P* = 0.057]. The two distinct imaging profiles between LAA and CE stroke subtypes may explain the discrepant time-dependent benefit of EVT observed in these two subtypes. Additional analysis restricting patients with pure LVO yielded similar results (Supplementary Tables 4, 5).

## Discussion

In this study, we report our single-center analysis of patients with LVOs of anterior circulation undergoing EVT within 24 h after time LKN. We demonstrate that in a real-world clinical setting where the LAA subtype accounted for 42% of all patients and groin puncture time was mostly delayed, EVT could still be effectively performed with a 41% functional independence rate at 90 days. The time-dependent benefit of EVT was observed in patients treated within 6 h after onset and in patients with CE-related stroke, rather than in the overall population and in patients with LAA-related stroke. Our study highlights the discrepancy in the time-dependent benefit of EVT stratified by stroke etiology.

Of note, 41% of patients in our cohort achieved 90-day functional independence (mRS of 0–2), which is within the range of 38–56% functional independence rate reported in other real-world registries with an extended therapeutic time window (Zi et al., 2017; Jansen et al., 2018; Huo et al., 2019; Jahan et al., 2019; Flottmann et al., 2021; Jia et al., 2021). However, the rate of functional independence was lower than that in other Asian cohorts (Zi et al., 2017; Huo et al., 2019; Jia et al., 2021). Meanwhile, the mortality rate and sICH rate were much higher than those in Endovascular Therapy for Acute Ischemic Stroke Trial (EAST) (Huo et al., 2019) and another Chinese nationwide registry (Jia et al., 2021). This may be in part due to an elderly population in our cohort with a median age of 71 years as opposed to 62–65 years in others (Zi et al., 2017; Huo et al., 2019; Jia et al., 2021). Apart from that, a significant treatment delay was observed in our cohort. This delay was even more obvious when compared with Western registries (Jansen et al., 2018; Jahan et al., 2019), whereas the rate of functional independence was comparable. Since no remarkable difference regarding age or stroke severity was noticed among our cohort and the Western registries, one possible explanation could be the different stroke subtype compositions across ethnicity and the less pronounced time effect in patients with LAA-related stroke.

Intracranial atherosclerosis is a major cause of ischemic stroke in Asian populations (Kim and Kim, 2014). In line with the Endovascular Treatment for Acute Anterior Circulation Ischemic Stroke Registry (ACTUAL) (Hao et al., 2017), our study demonstrated a 42% of patients classified as LAA subtype, while LAA only accounts for 19% in the German Stroke registry (Flottmann et al., 2021) and 13% in a large-scale Dutch registry (Boodt et al., 2020).

With a limited sample size, our study demonstrated a pronounced time-dependent benefit of EVT in patients with CE-related stroke, while no such association was observed in the overall cohort and in the subset of patients classified as LAA-related stroke. This is in accordance with one recent finding that patients with LAA have similar functional outcomes after EVT with CE patients despite a delay in symptom onset to recanalization (Lee et al., 2020). This discrepancy in the association between time and outcome may be explained by the robustness of collaterals and the resultant infarct growth in different subtypes. Along with other studies, our study showed that patients of the LAA subtype had significantly better collateral status (Zhang et al., 2018) and milder symptoms at presentation (Lee et al., 2020) than those of the CE subtype. Rather than a sudden occlusion in CE-related stroke, patients with LAA-related stroke suffer from a chronic period of steno-occlusive status and hemodynamic instability (Liebeskind et al., 2011). Previous studies (Lee et al., 2017) have demonstrated that angiogenic factors such as vascular endothelial growth factor (VEGF) were induced as a result of transient cerebral ischemia, building collateral pathways, thus improving collateral recruitment in patients of the LAA subtype. Our study further connected collateral status with both tissue outcome and clinical outcome. Accumulating evidence suggests that, besides a larger ischemic core at baseline (Chen et al., 2019), patients with poorer collateral status also have a faster infarct growth rate (Vagal et al., 2018; Lin et al., 2021), both of which have been reported to be independent predictors of poor clinical outcomes (Campbell et al., 2019; Chen et al., 2019). Similarly, by demonstrating distinct collateral profiles possessed by patients of CE subtype vs. those of LAA subtype, our study further found differences in core growth rate and final infarct volume in between. The status of collateral flow has been shown to modify the time-dependent benefit of EVT. Hwang et al. demonstrated that the OR of favorable outcome in patients with a poor collateral status significantly dropped as onset to reperfusion time or puncture to reperfusion time increased, while no such association was found in patients with good collaterals (Hwang et al., 2015). Shirakawa et al. (2021) previously reported the discrepancy in time-dependent benefit by stroke etiology in a Japanese Registry. However, without complete perfusion evaluation at baseline, the study was not able to explore further behind the phenomena. Assisted by detailed imaging analysis, our study was capable of validating the hypothesis and highlighted again the role of collateral status (Hwang et al., 2015; Vagal et al., 2018; Lin et al., 2021) in the fast-saving-brain track.

Furthermore, the association between earlier treatment and better clinical outcome was significant in patients punctured within 6 h from LKN, but this association became insignificant when including patients treated beyond 6 h from LKN. This is consistent with one single-center analysis (Snyder et al., 2020). Similar findings have also been reported in larger registries (Jahan et al., 2019; Nogueira et al., 2022), with a rapid loss of EVT benefit with treatment delay in the initial hours, transitioning to a slower loss of benefit in the periods later. Early after onset, regardless of infarct growth rate, most patients could have a small to moderate infarct core unrestricted by imaging criteria, while later after onset, patients with poor collaterals and fast infarct growth rate could proceed with large infarcts inappropriate for further intervention and were thus excluded. In other words, the remaining patients who received EVT in the late time window were selected to be with better collaterals and slower infarct growth, attenuating the association between LPT with outcomes. This is also reflected by the temporal distribution of different stroke subtypes. With less robust collaterals and potentially faster infarct growth, CE-related stroke presenting in the late time window was less likely selected out and only accounted for 35% of strokes beyond 6 h after LKN in contrast to 58% in the early 6-h time window.

Despite not being selected as an independent predictor for functional outcome in the overall population, LKN to puncture within 6 h was associated with a lower rate of ICH, which stood as an independent predictor for poor functional outcome. Similar findings have been reported in a pooled analysis of RCTs (Raychev et al., 2020) as well as registry studies (Hao et al., 2017; Jahan et al., 2019), reflecting the increased vulnerability to reperfusion injury with prolonged ischemia (Raychev et al., 2020). These findings serve as a strong reminder that rapid treatment should always be pursued even if time-dependent benefit is less pronounced in the LAA subtype.

Our study has several limitations. First, the data reported in this study are from a single-center endovascular database with a limited sample size. It is thus possible that with a larger sample size, the time-dependent benefit from EVT in the LAA subtype would be identified. Further validation in a larger cohort is warranted. Second, there was missing of perfusion imaging data and follow-up imaging, which accounted for 16% (23/140) either due to the absence of imaging scan or reconstruction failure. Nonetheless, the primary study cohort and the sensitivity analysis with complete perfusion data demonstrated similar results.

## Conclusion

Our single-center cohort of EVT with LVO of anterior circulation has a distinct composition of stroke subtypes in comparison with Western registries, with a higher proportion of patients with LAA-related stroke. Delay of groin puncture has a more pronounced effect on functional outcome in patients of CE subtype than those of LAA subtype. Reducing treatment delay is of great importance in improving the prognosis of patients after EVT, especially in those of CE subtype, and reducing the incidence of any ICH in all patients.

## Data availability statement

The data that support the findings of this study are available from the corresponding authors upon reasonable request.

## Ethics statement

The studies involving human participants were reviewed and approved by the Huashan Ethics Committee. The patients/participants provided their written informed consent to participate in this study.

## Author contributions

LH, XC, and QD contributed to conception and design of the study. LY and SL organized the database. YZ, LH, and YL assessed the imaging. YZ performed the statistical analysis and wrote the original draft. LH and XC reviewed and edited the draft. XC and QD supervised the whole investigation. All authors have read and approved the submitted manuscript.

## Abbreviations

EVT: endovascular treatment
LKN: last known normal
LAA: large artery atherosclerosis
LVO: Large Vessel Occlusion
LPT: last known normal to groin puncture time
CE: cardioembolic
mTICI: modified thrombolysis in cerebral ischemia scale
mRS: modified rankin scale
NIHSS: National Institute of Health Stroke Scale
NCCT: non-contrast CT
ICH: intracranial hemorrhage
sICH: symptomatic intracranial hemorrhage.
