# Supplementary material for: Association of time to groin puncture with patient outcome after endovascular therapy stratified by etiology
Source: Front Aging Neurosci. 2022 Oct 10;14:884087. doi: 10.3389/fnagi.2022.884087 (PMC9590449; doi:10.3389/fnagi.2022.884087)
Supplement: Supplementary file 1 [file Data_Sheet_1.docx]

Supplementary Material

[**Supplemental Methods**](#methods)

[**Figure S1.**](#fig) Study flow diagram

[**Table S1.**](#tab1) Characteristics and outcomes of patients with and without functional independence at 90 days

[**Table S2.**](#tab2) Predictors of any ICH without and with imaging variables included in patients with large vessel occlusion or severe (≥70%) stenosis

[**Table S3.**](#tab3) Predictors of functional Independence (mRS 0-2) at 90 days with imaging variables included in patients with large vessel occlusion or severe (≥70%) stenosis

[**Table S4.**](#tab4) Predictors of any ICH without and with imaging variables included in patients with pure large vessel occlusion

[**Table S5.**](#tab5) Predictors of functional Independence (mRS 0-2) at 90 days without and with imaging variables included in patients with pure large vessel occlusion

# Supplemental Methods

## Patient Selection for EVT

Candidacy for EVT is determined by an absence of a large territory hypodensity on NCCT, a presence of a large vessel occlusion or severe stenosis(≥70%) on CTA and a perfusion mismatch (volume of hypoperfused lesion/core volume>1.2). For patients with a baseline core volume over 70 ml, the eligibility for EVT was dependent upon the comprehensive judgement of the acute stroke team taking the demographic and clinical characteristics into consideration. Previously, only patients arriving within 6 hours after LKN were considered for EVT. With the successive release of trial results from DAWN (DWI or CTP Assessment with Clinical Mismatch in the Triage of Wake-Up and Late Presenting Strokes Undergoing Neurointervention with Trevo) and DEFUSE3 (The Endovascular Therapy Following Imaging Evaluation for Ischemic Stroke) in 2018, the treatment time window had thereafter been extended to 24 hours since LKN. Eligible patients were treated with intravenous thrombolysis according to the most updated Chinese guidelines.

## Endovascular Treatment

Endovascular intervention consisted of arterial catheterization with a micro-catheter to the level of occlusion, followed by thrombus aspiration or stent thrombectomy (Solitaire^TM^) or a combination of both. And in cases of vessel tortuosity, spontaneous recanalization or unstable vital signs, a simple catheterization and angiography without further treatment would be performed. Rescue therapy including balloon angioplasty, permanent stent and intra-arterial tirofiban was allowed and was left to the discretion of the intervention team.

# Supplemental Figure

**Figure S1.** Study flow diagram


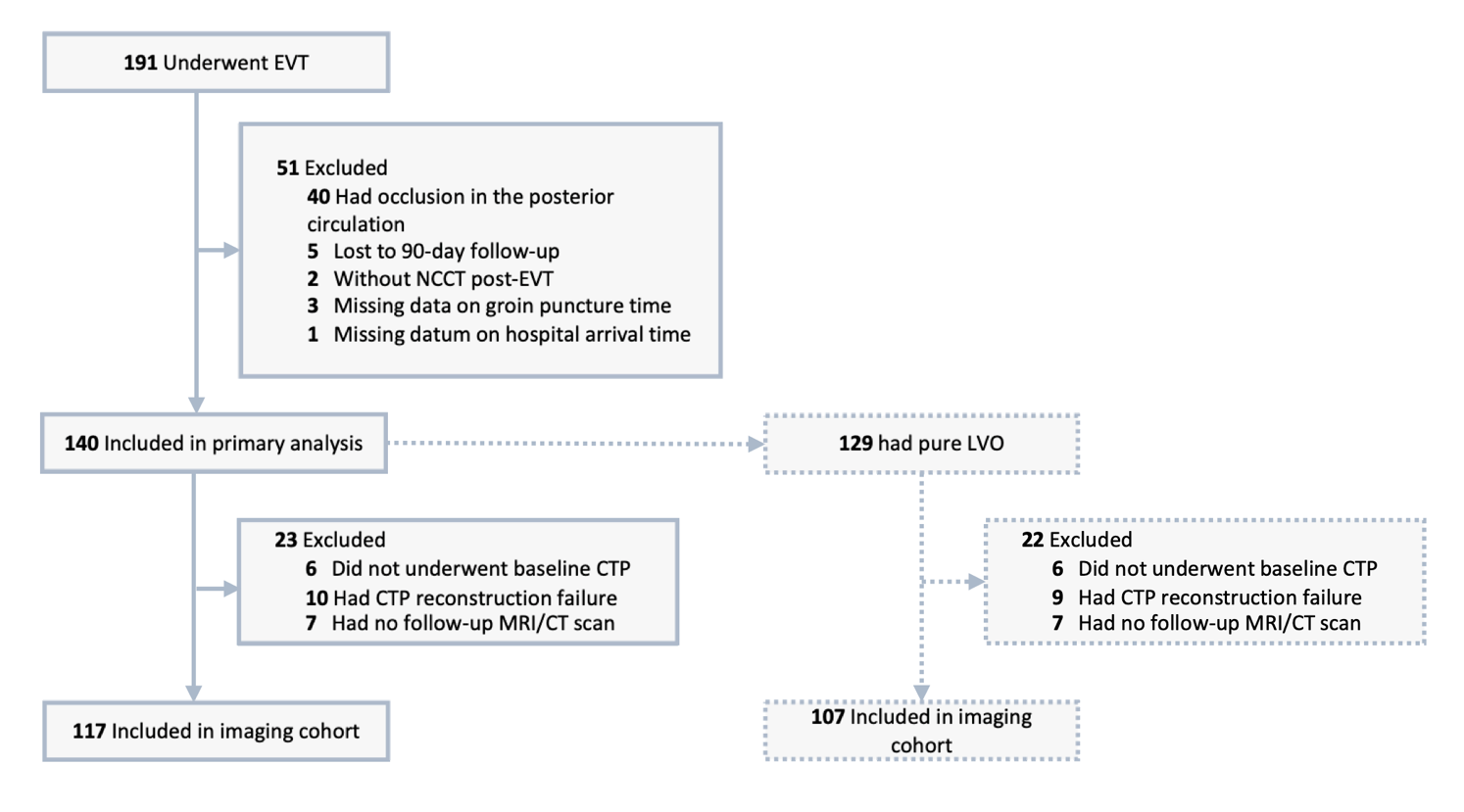


†Eleven out of 140 patients included in the primary analysis had severe stenosis in the anterior circulation, 9 of which were classified as LAA-related. Sensitivity analyses were performed both in patients with complete imaging profile and in patients with pure large vessel occlusion.

EVT, endovascular treatment; NCCT, non-contrast CT; CT, perfusion CT; LVO, large vessel occlusion

# Supplemental Tables

**Table S1.** Characteristics and outcomes of patients with and without functional independence at 90 days

|  | **mRS 3-6**  **(n=83)** | **mRS 0-2**  **(n=57)** | **P Value** |
| --- | --- | --- | --- |
| **DEMOGRAPHICS** |  |  |  |
| Age, median (IQR) | 74 (64, 81) | 67 (57, 74) | **0.001** |
| Female | 34 (41%) | 20 (35%) | 0.48 |
| NIHSS, mean (SD) | 16 (6) | 14 (5) | 0.06 |
| **MEDICAL HISTORY** | | | |
| Smoking | 28 (35%) | 20 (36%) | 0.83 |
| Hypertension | 54 (65%) | 33 (58%) | 0.39 |
| Atrial Fibrillation | 33 (40%) | 20 (35%) | 0.58 |
| Diabetes Mellitus | 27 (33%) | 13 (23%) | 0.21 |
| Stroke | 19 (23%) | 10 (18%) | 0.44 |
| Antiplatelet | 15 (18%) | 8 (14%) | 0.53 |
| Statin | 7 (8%) | 6 (11%) | 0.68 |
| **TOAST** |  |  | 0.10 |
| LAA | 30 (36%) | 29 (51%) |  |
| CE | 34 (41%) | 22 (39%) |  |
| Others† | 19 (23%) | 6 (11%) |  |
| **IMAGING FEATURES** | | | |
| Occlusion site |  |  | 0.88 |
| M1 | 51 (61%) | 34 (60%) |  |
| M2/ACA | 5 (6%) | 5 (9%) |  |
| ICA | 20 (24%) | 12 (21%) |  |
| Tandem | 7 (8%) | 6 (11%) |  |
| **TREATMENT DETAILS** |  |  |  |
| General Anesthesia | 44 (53%) | 29 (51%) | 0.80 |
| IVT | 35 (42%) | 26 (46%) | 0.69 |
| LDT (min), median (IQR) | 195 (70, 322) | 149 (82, 287) | 0.98 |
| DPT (min), median (IQR) | 164 (133, 190) | 150 (109, 191) | 0.24 |
| LPT (min), median (IQR) | 350 (240, 490) | 320 (222, 435) | 0.38 |
| LPT within 6h | 43 (52%) | 36 (63%) | 0.18 |
| **TECHNICAL EFFICACY** |  |  |  |
| mTICI>=2b | 46 (55%) | 47 (82%) | **<0.001** |
| **OUTCOME** |  |  |  |
| Any ICH | 44 (53%) | 10 (18%) | **<0.001** |
| sICH-ECASS-II | 18 (22%) | 0 (0%) | **<0.001** |
| 90d mortality | 30 (36%) | 0 (0%) | **<0.001** |

† Others include stroke of other determined aetiology and stroke of undetermined aetiology.

LDT, last known normal to hospital arrival time; LPT, last known normal to puncture time; DPT, door to puncture time.

**Table S2**. Predictors of any ICH with and without imaging variables included in patients with large vessel occlusion or severe (≥70%) stenosis

|  | **Without Imaging Variables (n=140)** | |  | **With Imaging Variables (n=117)** | |
| --- | --- | --- | --- | --- | --- |
|  | **OR (95% CI)** | **P value** |  | **OR (95% CI)** | **P value** |
| Stent retriever | 4.08 (1.69, 9.84) | 0.002 |  | 3.84 (1.29, 11.39) | 0.015 |
| LPT within 6h | 0.36 (0.17, 0.75) | 0.007 |  | 0.39 (0.16, 0.93) | 0.035 |
| FIV (ml) | - | - |  | 1.01 (1.00, 1.02) | <0.001 |

OR, odds ratio; CI, confidence interval; LPT, time from last known normal to groin puncture; FIV, final infarct volume

**Table S3**. Predictors of functional Independence (mRS 0-2) at 90 days with imaging variables included in patients with large vessel occlusion or severe (≥70%) stenosis

|  | **OR (95% CI)** | **P value** |
| --- | --- | --- |
| **All patients (n=117)** |  |  |
| Age | 0.92 (0.88, 0.96) | <0.001 |
| mTICI≥2b | 3.57 (1.14, 11.18) | 0.029 |
| Any ICH | 0.13 (0.04-0.48) | 0.002 |
| FIV (ml) | 0.99 (0.98, 1.00) | 0.003 |
| **Patients punctured within 6h after LKN (n=65)** | | |
| Age | 0.89 (0.83-0.96) | 0.002 |
| Any ICH | 0.05 (0.01-0.49) | 0.001 |
| FIV (ml) | 0.97 (0.95-0.99) | 0.002 |
| DPT† | 0.78 (0.64-0.94) | 0.011 |
| Dyslipidemia | 0.09 (0.01, 0.82) | 0.033 |
| **LAA patients (n=53)** | | |
| Age | 0.90 (0.84, 0.96) | 0.001 |
| Any ICH | 0.09 (0.02, 0.51) | 0.006 |
| **CE patients (n=46)** | | |
| Age | 0.74 (0.59-0.93) | 0.009 |
| Any ICH | 0.01 (0.00-0.54) | 0.023 |
| LPT† | 0.86 (0.76-0.98) | 0.019 |
| FIV (ml) | 0.96 (0.93-0.99) | 0.024 |

† Odds ratios are scaled per 10 minutes of delay in the listed interval

OR, odds ratio; CI, confidence interval; mTICI, modified Thrombolysis in Cerebral Ischemia Scale; ICH, intracranial Haemorrhage; FIV, final infarct volume; LKN, last known normal; NIHSS, National Institute of Health Stroke Scale; DPT, time from hospital arrival to groin puncture; LPT, time from last known normal to groin puncture

**Table S4**. Predictors of any ICH with and without imaging variables included in patients with pure large vessel occlusion

|  | **Without Imaging Variables (n=129)** | |  | **With Imaging Variables (n=107)** | |
| --- | --- | --- | --- | --- | --- |
|  | **OR (95% CI)** | **P value** |  | **OR (95% CI)** | **P value** |
| Stent retriever | 3.39 (1.31, 8.75) | 0.012 |  | - | - |
| LPT within 6h | 0.32 (0.15, 0.70) | 0.004 |  | 0.40 (0.17, 0.95) | 0.038 |
| FIV (ml) | - | - |  | 1.01 (1.00, 1.02) | 0.001 |

OR, odds ratio; CI, confidence interval; LPT, time from last known normal to groin puncture; FIV, final infarct volume

**Table S5**. Predictors of functional Independence (mRS 0-2) at 90 days without and with imaging variables included in patients with pure large vessel occlusion

|  | **Without Imaging Variables** | |  | **With Imaging Variables** | |
| --- | --- | --- | --- | --- | --- |
|  | **OR (95% CI)** | **P value** |  | **OR (95% CI)** | **P value** |
| **All Patients** | **N=129** | |  | **N=107** | |
| Age | 0.94 (0.91-0.98) | 0.001 |  | 0.92 (0.88-0.97) | <0.001 |
| mTICI≥2b | 3.25 (1.30-8.13) | 0.012 |  | 3.59 (1.13-11.43) | 0.03 |
| Any ICH | 0.12 (0.05-0.33) | <0.001 |  | 0.14 (0.04-0.53) | <0.001 |
| FIV (ml) | - | - |  | 0.99 (0.98-1.00) | <0.001 |
| **Patients Punctured within 6h after LKN** | **N=73** | |  | **N=60** | |
| Age | - | - |  | 0.91 (0.85-0.97) | 0.01 |
| Baseline NIHSS | 0.88 (0.78-0.99) | 0.039 |  | - | - |
| Any ICH | 0.07 (0.01-0.30) | <0.001 |  | 0.10 (0.01-0.70) | 0.02 |
| mTICI≥2b | 4.23 (1.14-15.72) | 0.032 |  | - | - |
| DPT† | 0.84 (0.73-0.96) | 0.009 |  | 0.78 (0.65-0.94) | 0.01 |
| FIV (ml) | - | - |  | 0.97 (0.96-0.99) | <0.001 |
| **LAA Patients** | **N=49** | |  | **N=43** | |
| Age | 0.91 (0.86-0.97) | 0.003 |  | 0.91 (0.86-0.97) | 0.01 |
| Any ICH | 0.24 (0.06-0.98) | 0.046 |  | - | - |
| FIV (ml) | - | - |  | 0.99 (0.97-1.00) | 0.05 |
| **CE Patients** | **N=56** | |  | **N=46** | |
| Age | 0.85 (0.77-0.95) | 0.004 |  | 0.74 (0.59-0.93) | 0.009 |
| Any ICH | 0.01 (0.00-0.19) | 0.002 |  | 0.01 (0.00-0.54) | 0.023 |
| LPT† | 0.90 (0.82-0.98) | 0.013 |  | 0.86 (0.76-0.98) | 0.019 |
| Diabetes | 0.08 (0.01-0.69) | 0.021 |  | - | - |
| FIV (ml) | - | - |  | 0.96 (0.93-0.99) | 0.024 |

† Odds ratios are scaled per 10 minutes of delay in the listed interval

OR, odds ratio; CI, confidence interval; mTICI, modified Thrombolysis in Cerebral Ischemia Scale; ICH, intracranial Haemorrhage; FIV, final infarct volume; LKN, last known normal; NIHSS, National Institute of Health Stroke Scale; DPT, time from hospital arrival to groin puncture; LPT, time from last known normal to groin puncture.
